# Tuning Shinkarev’s Bicycle: Separating the Parallel Cycles of Photosystem II Using Empirical Wavelet Transform

**DOI:** 10.3390/plants15040625

**Published:** 2026-02-16

**Authors:** Nicholas Ferrari, Brandon P. Russell, David J. Vinyard

**Affiliations:** Department of Biological Sciences, Louisiana State University, Baton Rouge, LA 70803, USA; nferr11@lsu.edu (N.F.); russeb7@rpi.edu (B.P.R.)

**Keywords:** photosystem II, oxygen-evolving complex, Joliot–Kok model, empirical wavelet transform

## Abstract

The oxygen-evolving complex (OEC) of Photosystem II (PSII) catalyzes light-driven water oxidation, a process necessary to sustain Earth’s atmospheric oxygen. Oxygen yields measured during single-turnover flash sequences exhibit period-four oscillations, which form the basis of the Joliot–Kok (S-state) model. However, when the oscillations of other processes contribute to the measured oxygen yield, fitting methods can conflate these signals and distort estimates of inefficiencies and initial S-state populations. To address this, we applied the empirical wavelet transform (EWT) as a model-independent method to separate overlapping oscillators and capture damping dynamics that are not well represented in Fourier analysis. We tested this framework on polarographic flash-oxygen traces from both our *Synechocystis* sp. PCC 6803 thylakoid membrane preparations and archival datasets on *Chlorella* and isolated chloroplasts. EWT consistently resolves the expected period-four component alongside a distinct binary oscillation. Simulations suggest that fitting this isolated period-four signal recovers VZAD parameters more accurately than analysis of raw traces, yielding different estimates for S-state distributions and transition probabilities. Notably, this binary oscillation aligns closely with semiquinone dynamics predicted solely from period-four fit parameters. These findings indicate that EWT can effectively distinguish complex signals in oxygen evolution, offering a framework potentially applicable to other spectroscopic probes of the S-state cycle.

## 1. Introduction

Oxygenic photosynthesis sustains the biosphere by converting solar energy into stored chemical potential. This process relies on the water–plastoquinone oxidoreductase activity of Photosystem II (PSII), which coordinates the four-electron oxidation of water with the two-electron reduction of a mobile quinone, Q_B_. Embedded within PSII is the oxygen-evolving complex (OEC), a Mn_4_CaO_5_(H_2_O)_4_ cluster [1]. This cluster is coupled to photo-oxidation of the dimeric chlorophyll primary donor, P_680_, via the redox-active Tyrosine-Z residue. Light-driven charge separation initiates forward electron transfer toward Q_B_ and results in the generation of the cation radical P_680_^+^. This cation radical acts as a powerful oxidant to extract electrons from the OEC, advancing it through five redox states (S_0_–S_4_). Through this cycle, the OEC accumulates the oxidizing equivalents required to split water and produce molecular oxygen.

The architecture of the OEC is an excellent example of evolutionary conservation. Since the emergence of oxygenic photosynthesis, the fundamental structure of the Mn_4_CaO_5_(H_2_O)_4_ cluster has remained unchanged across all oxygenic lineages, spanning from unicellular cyanobacteria to complex angiosperms [1,2,3]. The universality of this structure implies that the mechanism of water oxidation imposes strict constraints on the enzyme, preserving the geometry of the catalytic core. Consequently, the kinetic features observed in flash yield experiments are not unique to specific organisms but are signatures of the conserved machinery for water oxidation. Therefore, the kinetic insights gained from model organisms are directly translatable to the physiology of economically relevant plant species. Resolving these signals provides insight into the optimized, but constrained, solution that nature evolved to extract electrons from water.

To resolve the kinetics of this cycle, flash oxygen yield measurements monitor OEC turnover by advancing the S-states in discrete steps [4,5,6]. Polarographic recordings across a sequence of single-turnover flashes show a period-four oscillation of oxygen release, which is the foundation of the Joliot–Kok model [7]. In practice, the period-four pattern is not purely periodic because experimental traces of oxygen yield damp across successive flashes. This damping occurs because OEC turnover becomes progressively desynchronized [8,9].

Standard analyses fit S-state models directly to raw flash oxygen yield sequences to infer the initial S-state distribution and quantify turnover inefficiencies for a given set of conditions [10]. In addition to the original period-four cycle [11], extended models describe single advancements as hits (γ) and incorporate parameters for specific inefficiencies: misses (α) where centers do not advance, double-hits (β) where centers advance two states, and backward transitions (δ) where centers revert to the previous state. To account for signal loss, an inactivation parameter (ε) describes the fraction of centers that exit the catalytic cycle into a non-functional state (S_ε_) [12,13].

Since the discovery of period-four oscillations in flash oxygen by Joliot [4], consistent period-four oscillations have been observed using diverse spectroscopic probes. These include variable chlorophyll fluorescence [14,15], UV absorbance [16], magnetic resonance [17], X-ray absorbance [18] and other spectroscopies [19,20,21]. Collectively, these datasets establish that period-four oscillations arise specifically from redox intermediates of the OEC.

Raw flash oxygen yields are complex because the donor and acceptor sides of PSII operate with different periodicities. While the OEC cycles through four oxidation steps to release oxygen, the acceptor side cycles through two steps. Consequently, the semiquinone intermediate (Q_B_^−^) accumulates on odd-numbered flashes and is fully reduced to plastoquinol (Q_B_H_2_) on even-numbered flashes. The phase relationship between acceptor and donor cycles depends on the initial state of the OEC. Following dark adaptation, approximately 75% of centers are in S_1_ and approximately 25% are in S_0_. As described by Shinkarev and Wraight, this difference results in a dominant oscillator (Cycle V) that is most likely to evolve oxygen on the third flash and a minor oscillator (Cycle W) that is most likely to evolve oxygen on the fourth flash [22].

Period-two oscillations have been detected and arise from interactions between a pool of redox-active intermediates and PSII [6,23,24]. The mechanistic interpretation for period-two oscillations is unclear and is argued to come from either quinone reduction [25] or superoxide formation [24]. Shinkarev provided a simple model for period-two oscillations dependent on the presence of Q_B_^−^. For both Cycles V and W, Q_B_^−^ is present following successful hits of odd-numbered flashes (*n*) assuming γ > 0.5 (Equation (1)) [22,26].

Q_B_^−^ = 0.5 [1 − (1 − 2γ)^n^] (1)

When Q_B_^−^ is present on the acceptor side of PSII, charge recombination between Q_A_^−^ and the donor side is more likely [26,27]. Depending on the frequency of single turnover flash application, this reaction gives rise to a Joliot–Kok model miss [28]. The equilibrium constant and rate of charge recombination depends on the specific S-state. Therefore, while Cycles V and W are synchronized in terms of the presence of Q_B_^−^, they are predicted to vary in terms of miss probability per flash number.

Fourier analysis is used to identify oscillators in flash oxygen yield data by transforming the signal into the frequency domain where the dominant frequencies appear as peaks [25,27]. However, this method relies on decomposing the data into sine waves, fixed basis functions that extend with constant amplitude across the entire flash sequence. This implementation creates a conflict with experimental flash yield sequences, which damp rapidly. The resulting Fourier spectrum represents a static frequency profile that does not distinguish between a constant oscillation and one that decays rapidly. Consequently, peaks in the frequency domain can broaden or overlap upon Fourier transformation of the time series data, and the OEC-linked component of the signal may become merged with interfering dynamics.

The signal properties of flash oxygen yield are not uniform across the sequence. The balance of contributing processes shifts with each successive flash as the enzyme population desynchronizes. A standard Fourier spectrum cannot distinguish overlapping signals with unique damping profiles because it aggregates the dynamic behavior into a static summary. Therefore, we applied the empirical wavelet transform (EWT) as a model-independent decomposition method [29]. Unlike methods constrained by fixed basis functions, EWT partitions the frequency spectrum based on the signal’s own spectral boundaries. This adaptive approach allows us to isolate the OEC period-four component from overlapping transient interference. By separating these dynamics, EWT offers a clearer window into the underlying kinetics of water oxidation without the influence of acceptor-side cycling.

## 2. Results

### 2.1. Parameter Recovery in Simulated Bicycle Kinetics

#### 2.1.1. Simulation of Interacting Donor and Acceptor Oscillators

**Figure 1:**
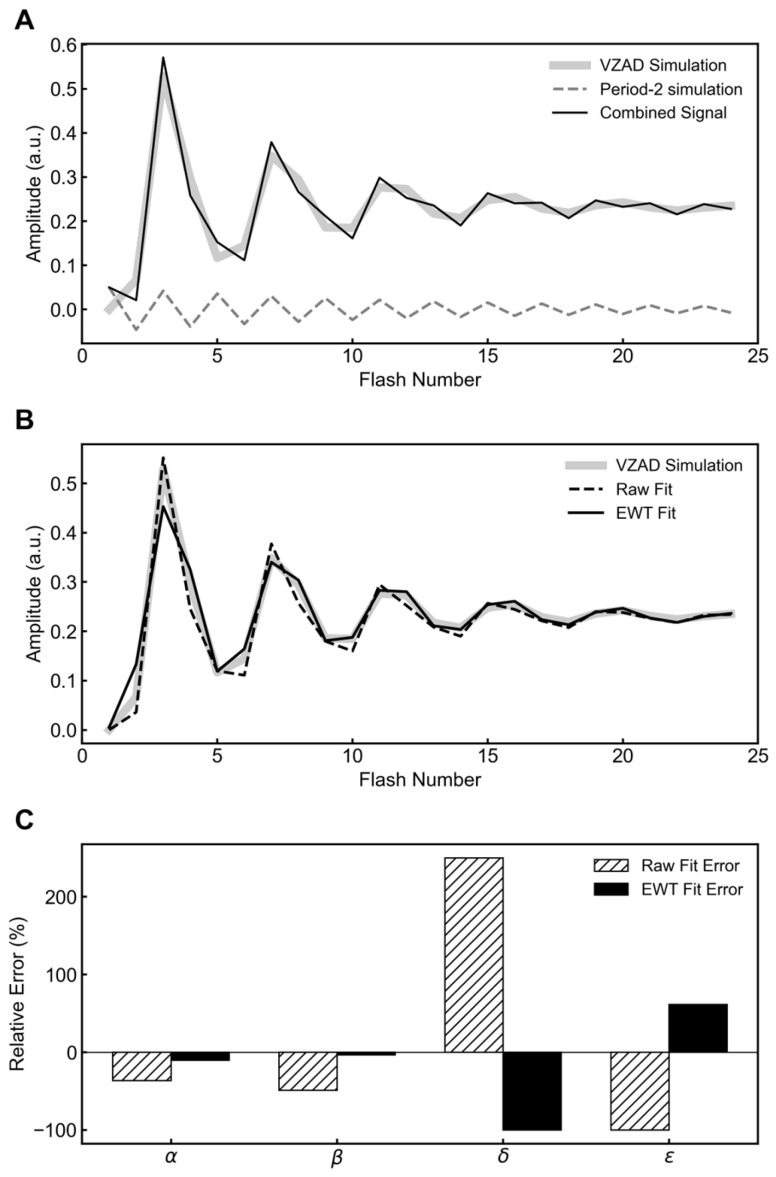
Validation of EWT-based VZAD parameter recovery with period-two interference. (**A**) The simulated biological signal (gray line) was generated with the parameters in Table 1. A decaying period-two oscillator was added to produce the combined signal (solid black line). (**B**) Comparison of the recovered oscillation sequence against the simulated signal (gray line). The raw fit (dashed black line) applied directly to the combined signal deviates from the simulated oscillator. The empirical wavelet transform (solid black line) separates the period-four oscillator prior to fitting, resulting in a reconstruction that more closely overlays the simulated signal. (**C**) Relative percentage error for the miss (α), double-hit (β), backward transition (δ), and inactivation (ε) parameters. The EWT method (solid black) reduces error across all parameters compared to the raw fit (hatched).

The ability of the EWT to recover accurate S-state parameters was first evaluated using simulated flash yield sequences mixed with simulated period-two interference. Extended Joliot–Kok model parameters were chosen to simulate an efficient system as presented in Table 1. The chosen parameters result in 75% Cycle V and 25% Cycle W. A dampened binary oscillator was simulated as shown in Figure 1A. The two functions were combined by addition to represent raw experimental data.

#### 2.1.2. Comparative Accuracy of S-State Parameter Fitting

The combined signal was fit using the VZAD model (Figure 1 and Table 1). Next, the combined signal was subjected to EWT filtration. A period-four component was extracted which was independently fit using the VZAD model (Figure 1 and Table 1).

The simulated Joliot–Kok oscillator has a miss parameter (α) of 0.100. When the combined function was modeled using VZAD, the best fit for α was 0.063. Following EWT filtration, a closer fit of 0.090 was obtained. Fitting of the combined function also underestimated double hits (β) and inactivations (ε). Backward transitions (δ) were poorly captured in both approaches. These differences are presented in Figure 1C in terms of relative error. Overall, the VZAD fit to the EWT-derived function data provided a more accurate retrieval of kinetic parameters compared to the VZAD fit to the raw combined data.

### 2.2. Spectral Isolation and Identification of Acceptor Side Kinetics

#### 2.2.1. Decomposition of Flash Oxygen Yields in *Synechocystis*

**Figure 2:**
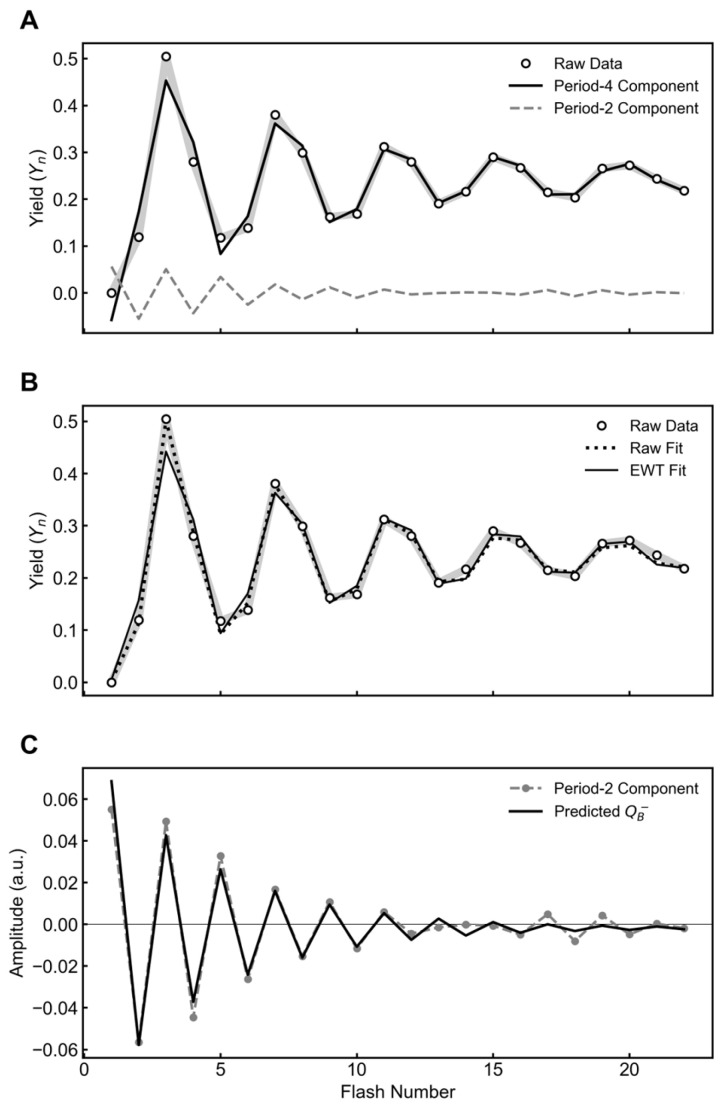
Separation of donor-side and acceptor-side oscillation in flash oxygen yields. Polarographic oxygen yields were measured from *Synechocystis* sp. PCC 6803 thylakoid membranes (30 µg chlorophyll) supplemented with PPBQ and ferricyanide, illuminated with single-turnover flashes at 1 Hz. (**A**) EWT decomposition separates the raw signal (raw data, circles) into a period-four oscillation (period-4 component, solid black line) and a period-two component (period-2 component, dashed gray line). (**B**) VZAD model fitted directly to the raw data (raw fit, dotted line) and to the EWT-isolated period-four component (EWT fit, solid line). (**C**) The isolated period-two component is overlaid with the predicted population of the semiquinone state (predicted Q_B_^−^, solid black line), calculated using the kinetic parameters derived from the period-four EWT fit.

Turning to experimental data, a typical flash oxygen pattern from *Synechocystis* sp. PCC 6803 thylakoid membranes is presented in Figure 2A. This raw data was fit using the VZAD model and presented in Figure 2B. Separately, this raw data was filtered using EWT and period-four and period-two components were extracted (Figure 2A). The period-four component was then fit using the VZAD model (Figure 2B and Table 2).

In this dataset, a low-frequency period-four oscillator and high-frequency period-two oscillator are routinely extracted from raw flash oxygen data. The period-four component clearly represents OEC cycling and the Joliot–Kok model.

#### 2.2.2. Comparison of Period-Two Oscillations to Semiquinone Dynamics

To understand the biological basis of the period-two component extracted using EWT, we compared the data to a modeled prediction of the Q_B_^−^ population (Equation (1)) [22]. This theoretical prediction was generated using the kinetic parameters derived empirically from the VZAD fit of the period-four component. As shown in Figure 2C, the isolated period-two signal is correlated with the predicted Q_B_^−^ cycle. The agreement in both oscillation phase and damping profile supports the idea that EWT isolates the acceptor-side pathway, enabling the retrieval of acceptor dynamics from flash oxygen experiments.

### 2.3. Robustness of EWT Across Datasets

To assess the applicability of this framework to more conditions, we applied the decomposition algorithm to archival datasets from other laboratories. This tested the method’s ability to distinguish the period-two signal without manual tuning.

#### 2.3.1. Analysis of Historical Data from *Chlorella*

**Figure 3:**
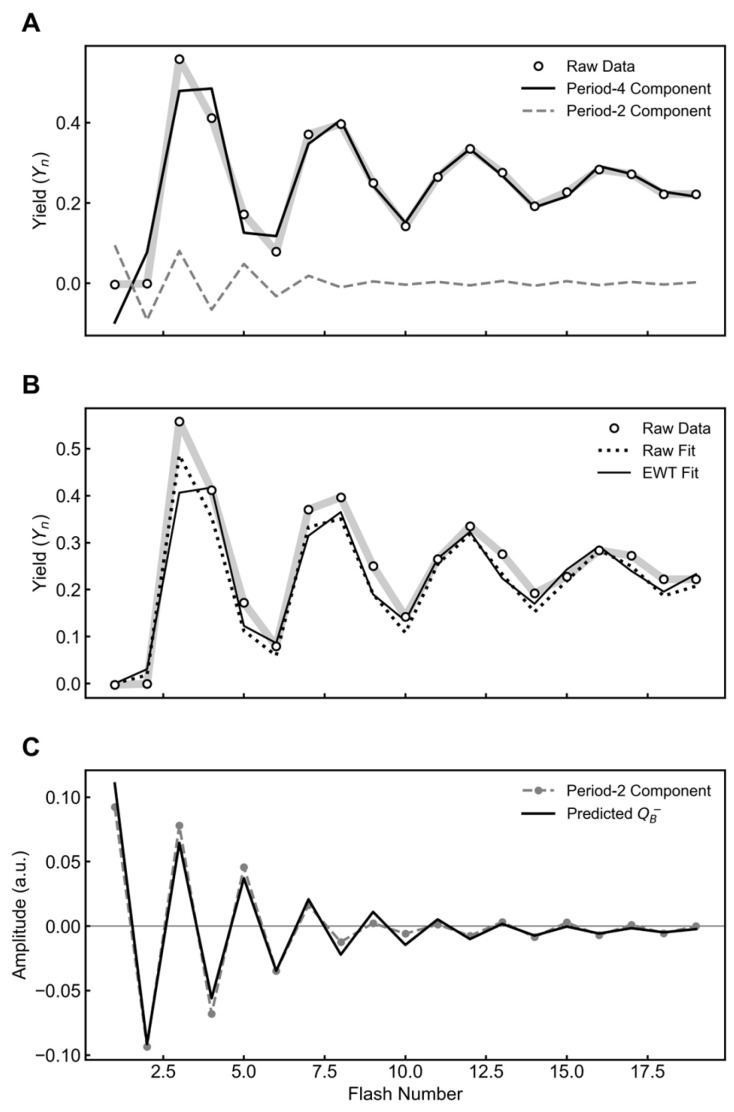
EWT decomposition and kinetic analysis of flash oxygen yields from *Chlorella*. (**A**) EWT decomposition separates the raw signal (raw data, circles) into a period-four oscillation (period-4 component, solid black line) and a period-two component (period-2 component, dashed gray line). (**B**) VZAD model fitted directly to the raw data (raw fit, dotted line) and to the EWT-isolated period-four component (EWT fit, solid line). (**C**) The isolated period-two component is overlaid with the predicted population of the semiquinone state (predicted Q_B_^−^, solid black line), calculated using the kinetic parameters derived from the period-four EWT fit.

We processed the flash oxygen yields from whole-cell preparations of *Chlorella* [4]. As shown in Figure 3, the raw signal is successfully resolved into a period-four and period-two oscillator. The initial S-state distribution for the EWT fit has higher S_0_ and lower S_1_ populations than the raw fit (Table 3). The shift of these populations is visualized through a lower oxygen yield on the third flash in Figure 3. The parameters were estimated by the same method described in Section 2.2.1.

#### 2.3.2. Analysis of Historical Data from *Spinacia*

To complement the whole-cell preparations, we also examined the flash oxygen yield sequence from the isolated chloroplasts of *Spinacia oleracea* [11]. The double-hit probability in this data set was identical (3.1%) between the raw and EWT-filtered fits, while the miss parameter decreased from 7.9% to 6.3% (Table 4). In contrast, the initial S-state distribution shifted significantly. The raw fit yielded an initial S_1_ population of 74%, while the EWT-filtered fit estimated 55% S_1_ and 38% S_0_ (Table 4).

**Table 4:** Fitted parameters from the VZAD model for data presented in Figure 4.

Parameter	Raw Fit	EWT Fit
Miss (α)	0.079	0.063
Double-hit (β)	0.031	0.031
Backward Transitions (δ)	0	0
Inactivation (ε)	0	0
Hits (γ)	0.890	0.906
[S_0_]	0.264	0.380
[S_1_]	0.736	0.549
[S_2_]	0	0.071
[S_3_]	0	0
[S_ε_]	0	0

**Figure 4:**
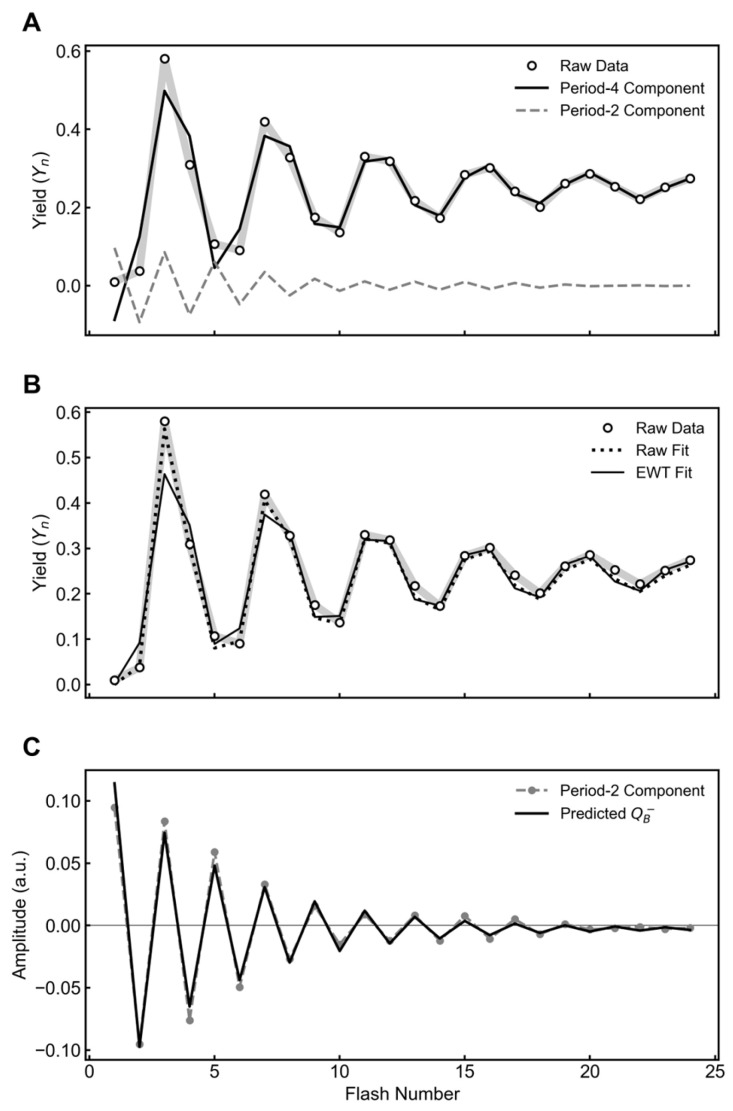
EWT decomposition and kinetic analysis of flash oxygen yields from *Spinacia oleracea*. (**A**) EWT decomposition separates the raw signal (raw data, circles) into a period-four oscillation (period-4 component, solid black line) and a period-two component (period-2 component, dashed gray line). (**B**) VZAD model fitted directly to the raw data (raw fit, dotted line) and to the EWT-isolated period-four component (EWT Fit, solid line). (**C**) The isolated period-two component is overlaid with the predicted population of the semiquinone state (predicted Q_B_^−^, solid black line), calculated using the kinetic parameters derived from the period-four EWT fit.

## 3. Discussion

### 3.1. Resolving the Bicycle Model with Empirical Wavelet Transform

Interpreting flash oxygen patterns from PSII requires considerations of both the electron donor and acceptor sides. Shinkarev and Wraight addressed this challenge by separating the population based on initial S-state, thus generating Cycles V and W. This analysis of a “bicycle” model of PSII turnover allowed them to account for binary deviations of raw signals resulting from the acceptor side [30].

Here, we present an alternative “bicycle” model in which distinct period-four and period-two signals are combined. The net result is very similar to the outcome of Shinkarev and Wraight’s analysis. However, this work, which relies on EWT, allows the two oscillators to be separated in a model-free approach.

In the framework of Multi-Resolution Analysis (MRA), which serves as the mathematical foundation for EWT, a signal is decomposed into independent subspaces [29]. These include an approximation space and a detail space. Mathematically, the approximation space is spanned by a scaling function which acts as a low-pass filter to capture the coarse, fundamental trends of the signal. This dominant component is the period-four oscillator in this application. The detail space is spanned by a wavelet function which acts as a band-pass filter to resolve fine, high-frequency fluctuations. This minor component is the period-two oscillator in this application. EWT constructs these filters adaptively. Local maxima on the Fourier spectrum are identified, and boundaries are established between the spectral troughs between them.

As shown in Figure 2C, the period-two component is strongly correlated with the predicted semiquinone population, matching both the oscillatory phase and the damping profile. To further verify this method, we applied the same decomposition to the seminal datasets of Joliot et al. (1969) and Forbush et al. (1971) [4,11]. In both cases, the extracted period-two mode exhibited the same characteristic correlation with the predicted acceptor dynamics (Figure 3 and Figure 4), supporting the effectiveness of this analysis across different experimental systems.

### 3.2. Mitigating Fitting Artifacts in S-State Analysis

The simulation results reveal an artifact inherent to fitting raw flash oxygen yield data: the possible conflation of acceptor dynamics with donor-side recombination. The VZAD model forces a single homogenous cycle onto a heterogenous signal, leading optimization algorithms to misinterpret the period-two oscillation as rapid damping. This leads to an overestimation of the backward transition probability and the introduction of artificially high initial S_2_ populations to compensate.

Fitting the EWT-filtered approximation space component effectively avoids this artifact. By removing the period-two interference before fitting, optimization algorithms recover the transition probabilities with higher accuracy. Although this process resulted in an underestimation of the backward transition, the overall deviation from the true value was lower than that of the raw fit. This result supports the idea that EWT acts as a filter for the donor-side pathway, preserving the core kinetic parameters even in the presence of significant period-two interference.

### 3.3. Advantages and Limitations of the in Silico Approach

The ability of EWT to separate oscillators offers possible advantages over traditional biochemical approaches. Isolating donor-side kinetics requires the use of exogenous electron acceptors or inhibitors to suppress acceptor-side feedback [28,31]. However, these treatments can alter the native physiological state of Photosystem II [32]. EWT offers an in silico alternative to recover S-state cycle parameters with minimal changes to the sample environment. This computational decoupling is particularly valuable for comparative studies of OEC function in diverse strains or mutants. Structural modifications to the environment surrounding the Mn_4_CaO_5_(H_2_O)_4_ cluster often result in complex phenotypes where donor-side inefficiencies are entangled with altered acceptor-side electron transfer [25]. Filtration with EWT allows for the assignment of kinetic defects specifically to the water-oxidizing machinery. This method can also be applied retroactively to analyze historical datasets, as demonstrated by our recovery of acceptor dynamics from the Joliot et al. and Forbush et al. flash sequences.

However, caution is warranted regarding parameter sensitivity. Our analysis revealed that the estimation of the initial S-state distribution depends heavily on the period-two oscillator, since this component contributes most significantly to the combined signal during the first flashes. Consequently, error in isolating these spectral modes can be amplified by the fitting algorithm, resulting in biased estimations of dark-adapted populations.

This sensitivity extends to the estimation of backward transitions, a parameter that remains challenging to resolve. Simulation results suggest that fitting VZAD parameters to the raw data conflates acceptor-side oscillations with rapid damping, leading to an overestimation of backward transitions. This likely occurs because the VZAD model imposes a single kinetic system on a heterogenous signal driven by both the OEC and the quinone pool. The optimization algorithm misinterprets the high-frequency (period-two) variance as accelerated de-synchronization. Therefore, solutions with an artificially high backward transition probability better approximate the rapid decay profile of the raw data. While EWT removes period-two interference, the original signal loses the high-frequency information, resulting in a smoother filtered signal. In this case, the optimization algorithm may attribute genuine backward transitions to misses, since both events can lead to similar de-phasing effects and often α > δ. For example, two consecutive misses can mathematically mimic the de-phasing effect of a backward transition followed by a hit (Equation (2)).

α(α(S_3_)) = γ(δ(S_3_)) (2)

Sensitivity also highlights a critical requirement for the proper application of EWT. The method assumes that donor-side (period-four) and acceptor-side (period-two) signals occupy separable frequency bands. In scenarios where the periodicities converge, the spectral peaks may overlap, inhibiting the data-driven boundary detection required for proper segmentation.

## 4. Materials and Methods

### 4.1. Thylakoid Membrane Preparation

Thylakoid membranes were isolated from wild-type *Synechocystis* sp. PCC 6803 as previously described and resuspended in a buffer containing 50 mM MES-NaOH (pH 6.3), 20 mM CaCl_2_, 5 mM MgCl_2_, 8 mM NaHCO_3_, 1.2 M betaine, and 10% (*v*/*v*) glycerol [33].

### 4.2. Polarographic Oxygen Yield Measurements

For flash oxygen measurements, a homebuilt electrode system (Pt_(s)_ cathode and Ag_(s)_ anode) was connected to a potentiostat (SP-50, BioLogic) [33]. Each sample contained thylakoids equivalent to 30 µg of chlorophyll, 250 µM *p*-phenyl-1,4-benzoquinone (PPBQ), 1 mM K_3_[FeCN_6_], and 3000 U of bovine catalase. Chronoamperometry was used to monitor oxygen yields during a series of single-turnover flashes supplied by a white LED flash lamp (35 µs flash duration delivered at 1 Hz).

### 4.3. Custom Flash Lamp

#### 4.3.1. Circuit Design

The LED driver circuit follows a design protocol previously utilized [34]. The circuit is powered by a DC voltage source (Korad KD6003D, Dongguan Korad Technology Co. Ltd., Dongguan, China) set to 36 V. A white-light LED with 130 W of power (Xlamp CXA2530, Cree LED, Durham, NC, USA) was configured in series with a 10 Ω carbon film resistor and in parallel with a diode (MUR160RLG, onsemi, Scottsdale, AZ, USA) to prevent reverse polarity. The current flow was controlled by a microcontroller (Arduino Uno R3, Arduino S.r.l., Monza, Italy) programmed to increase the gate potential of a MOSFET (IRFB3206PbF, Infineon Technologies AG, Neubiberg, Germany) to 5 V on a user-defined interval. The applied voltage on the MOSFET gate switches the LED circuit on until the microcontroller returns the gate potential to 0 V.

#### 4.3.2. Validation of Flash Duration

The LED flash durations were detected with a PIN photodiode (QSD2030, onsemi, Scottsdale, AZ, USA) configured with a reverse bias of 12 V. Light-induced current was converted into voltage by a transimpedance amplifier (LM358P, Texas Instruments, Dallas, TX, USA). Voltage was measured using an oscilloscope (GDS-1102A, Good Will Instrument Co. Ltd., New Taipei City, Taiwan). Each signal was numerically integrated using the composite trapezoidal rule (Figure S1).

### 4.4. Data Processing and Kinetic Modeling

#### 4.4.1. Archived Data Processing

Data from Joliot [4] and Forbush [11] were digitized using OriginPro version 2020.

#### 4.4.2. Empirical Wavelet Transform Implementation

Wavelet analyses were performed in Python 3.11.12 using the pyewt library available at https://github.com/jegilles/pyewt (accessed on 28 November 2025). Python scripts that implement empirical wavelet transform for flash oxygen data are available in Supplementary Materials S1 and S2. Generative AI tools (Google Gemini 3) were utilized to assist in the generation and refinement of the Python code used for data visualization (Figure 1 and Figure 2).

#### 4.4.3. VZAD Modeling

Raw and decomposed spectra were analyzed by the VZAD model to determine initial S state populations and inefficiency parameters [10]. In all fitting procedures, the S_3_ and S_ε_ populations were constrained to zero to reflect experimental observations of dark-adapted samples [19,35]. An updated version of the VZAD software in Python 3.11.12 is provided in Supplementary Materials S3. Generative AI tools (ChatGPT GPT-4o) were utilized to assist in the conversion and refinement of the Python code for VZAD simulations.

#### 4.4.4. Empirical Wavelet Transform Computation

Flash oxygen yield sequences are finite, discrete observation windows with asymmetric boundaries. One computational challenge arose from the difference between the signal endpoints because sequences started from a dark-adapted state and ended closer to steady-state. Spectral transforms assume the data is a continuous loop, where the last point returns to the value of the first point. Since the oxygen yield measured on the final flash does not return to the value measured on flash 1, an artificial step function is introduced. This instantaneous jump results in artificial high-frequency peaks. To mitigate this, we implemented a reflection padding protocol before EWT. The experimental signal was reflected to create a symmetric boundary that begins and ends at the same value.

The conditioned oxygen yield signal was then transformed into the frequency domain through a Fourier transform. This spectrum is partitioned by detecting local maxima and defining the boundaries as the local minima between them. The result is that the original signal is decomposed into independent bands of frequencies (modes) defined by spectral features.

We subtracted the mode with a peak frequency closest to a period-two cycle from the spectrum, excluding it from the signal reconstruction. The remaining modes were summed to produce the filtered period-four signal. The excluded period-two mode was reconstructed into its own signal for analysis.

The reconstructed period-two signal was compared to a predicted semiquinone population, which was simulated with kinetic parameters derived from the VZAD fit of the filtered period-four signal and Equation (1). The units of these signals are mismatched, with oxygen yield defined arbitrarily and predicted semiquinone population as a probability. To compare the dynamics, values were scaled to normalize differences in their respective units. Both sequences were centered with a mean equal to zero. The root mean square (RMS) of the simulated quinone population was scaled to the RMS of the experimental period-two signal. This resulted in an alignment of magnitude, allowing a direct evaluation of phase and damping profile.

## 5. Conclusions

The application of EWT to flash oxygen yield sequences is a useful tool in the kinetic analysis of PSII. The intrinsic heterogeneity described by the Shinkarev and Wraight “bicycle” model is addressed in a new way by EWT’s model-independent, data-driven separation of donor- and acceptor-side dynamics. This approach has been shown to improve the accuracy of the S-state parameter by isolating the signal from period-two interference. Demonstrated by retrospective analysis of historical datasets, EWT offers a framework for interpreting both new and archival data. Ultimately, the integration of multi-resolution signal processing is a clearer lens for dissecting the complex electronic mechanisms of water oxidation.

## Figures and Tables

**Table 1:** Fitted parameters from the VZAD model for data presented in Figure 1.

Parameter	Simulated Value	Raw Fit	EWT Fit
Miss (α)	0.100	0.063	0.090
Double-hit (β)	0.050	0.025	0.048
Backward Transitions (δ)	0.020	0.070	0
Inactivation (ε)	0.010	0	0.016
Hits (γ)	0.820	0.842	0.846
[S_0_]	0.250	0.219	0.339
[S_1_]	0.750	0.781	0.558
[S_2_]	0	0	0.103
[S_3_]	0	0	0

**Table 2:** Fitted parameters from the VZAD model for data presented in Figure 2.

Parameter	Raw Fit	EWT Fit
Miss (α)	0.075	0.066
Double-hit (β)	0.041	0.036
Backward Transitions (δ)	0.006	0
Inactivation (ε)	0	0
Hits (γ)	0.878	0.898
[S_0_]	0.276	0.334
[S_1_]	0.643	0.52
[S_2_]	0.081	0.146
[S_3_]	0	0
[S_ε_]	0	0

**Table 3:** Fitted parameters from the VZAD model for data presented in Figure 3.

Parameter	Raw Fit	EWT Fit
Miss (α)	0.098	0.081
Double-hit (β)	0.015	0.033
Backward Transitions (δ)	0	0
Inactivation (ε)	0	0
Hits (γ)	0.887	0.886
[S_0_]	0.336	0.500
[S_1_]	0.664	0.500
[S_2_]	0	0
[S_3_]	0	0
[S_ε_]	0	0

## Data Availability

The original contributions presented in this study are included in the article/Supplementary Materials. Further inquiries can be directed to the corresponding author.

## Supplementary Materials

The following supporting information can be downloaded at https://www.mdpi.com/article/10.3390/plants15040625/s1, Figure S1: The LED flash durations detected with a PIN photodiode (Onsemi QSD2030); Table S1: Oxygen yield sequences used to produce Figure 2, Figure 3 and Figure 4; Appendix S1: Simulation and recovery of parameters script for Figure 1 written in Python.; Appendix S2: Spectral isolation and quinone model script for Figure 2, Figure 3 and Figure 4 written in Python.; Appendix S3: Updated version of VZAD written in Python.
