# Supplementary material for: Tuning Shinkarev’s Bicycle: Separating the Parallel Cycles of Photosystem II Using Empirical Wavelet Transform
Source: Plants (Basel). 2026 Feb 16;15(4):625. doi: 10.3390/plants15040625 (PMC12944290; doi:10.3390/plants15040625)
Supplement: Supplementary file 1 [file plants-15-00625-s001.zip › plants-4104867-supplementary.pdf]

Supplementary Information

Tuning Shinkarev's Bicycle: Separating the Parallel Cycles of Photosystem II Using Empirical Wavelet Transform

Nicholas Ferrari†, Brandon P. Russell†, ‡ and David J. Vinyard*

Department of Biological Sciences, Louisiana State University, Baton Rouge, Louisiana 70803, USA; nferr11@lsu.edu (N.F.); russeb7@rpi.edu (B.P.R.)

* Correspondence: dvinyard@lsu.edu; Tel: +1 (225) 578-0958

† These authors contributed equally to this work.

‡ Present address: Department of Chemistry and Chemical Biology, The Baruch '60 Center for Biochemical Solar Energy Research, Rensselaer Polytechnic Institute, Troy, NY 12180, USA

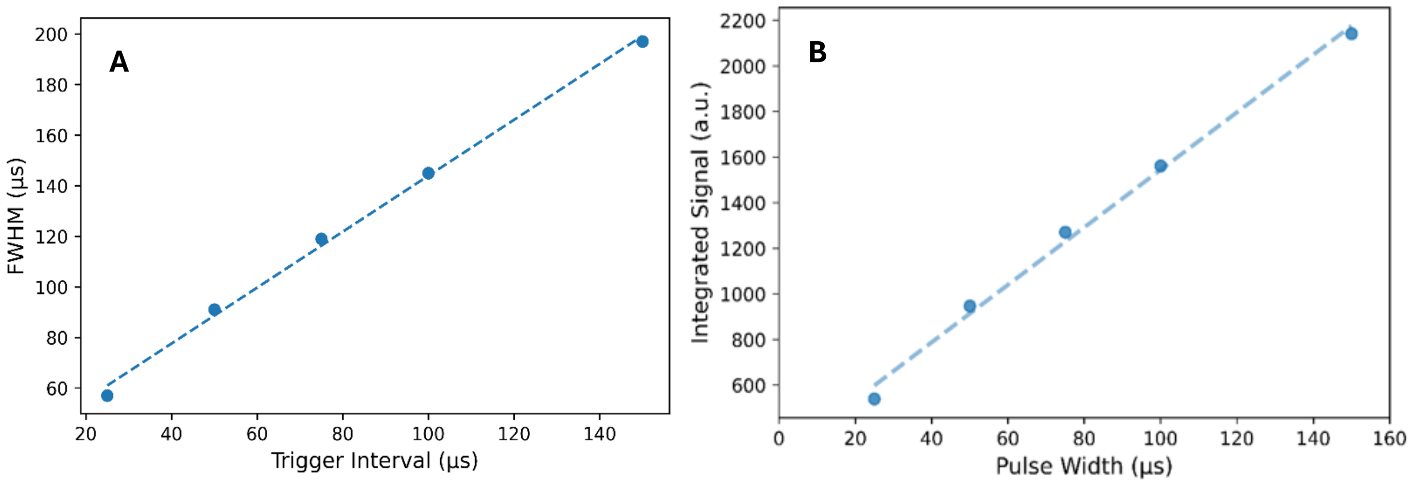

Figure S1. The LED flash durations detected with a PIN photodiode (Onsemi QSD2030). (A) Full width half max of the LED rise and decay as a function of the trigger interval. The dashed line denotes the line of best fit. (B) Numeric integration of the photodiode signal as a function of pulse width. The dashed line denotes the line of best fit.

Table S1. Oxygen yield sequences used to produce Figures 2-4.

Flash (n)	Forbush	This Study	Joliot
1	0.03831	0	-0.2523
2	0.14598	0.23659	-0.06534
3	2.25077	1	55.99389
4	1.20035	0.55521	41.27801
5	0.41471	0.23344	17.25453
6	0.35197	0.27445	7.94693
7	1.62557	0.75394	37.17839
8	1.27126	0.59306	39.78238
9	0.67902	0.32177	25.07985
10	0.52934	0.33438	14.27663
11	1.28076	0.6183	26.56213
12	1.2346	0.55521	33.63964
13	0.84298	0.37855	27.68384
14	0.67238	0.42902	19.29766
15	1.09992	0.57413	22.83642
16	1.16979	0.52997	28.43166
17	0.93485	0.42587	27.30994
18	0.78065	0.40379	22.27556
19	1.01234	0.52681	22.27556
20	1.10931	0.53943	
21	0.98108	0.48265	
22	0.85832	0.43218	
23	0.97568		
24	1.06337		

Appendix S1. Simulation and recovery of parameters script for Figure 1 written in Python.

```
# -*- coding: utf-8 -*-
"""
Created on Mon Dec 29 13:32:45 2025

@author: Nicholas
gemini3
VZAD Model + Padded EWT: Figure 1 Simulation
(Final Publication Style - Grayscale + Data Table)
"""

import numpy as np
import matplotlib.pyplot as plt
from scipy.optimize import minimize
import pandas as pd
import os

# Try/Except for pyewt
try:
  from pyewt.defaultparams import Default_Params
  from pyewt.ewt1d import ewt1d, iewt1d
except ImportError:
  print("Error: pyewt not found. Please install via: pip install pyewt")
  exit()

# ==============================================================
# 1. VZAD Kok Model Simulation
# ==============================================================
def simulate_VZAD_Y(params, steps):
  a, b, d, e, S0, S1, S2, S3, Se = params
  P = np.array([
    [a,          1 - a - b,     b,          0,          0],
    [0,          a,          1 - a - b,     b,          0],
    [b,          d,          a,          1 - a - b - d,   0],
    [1 - a - b - d - e, b,          d,          a,          e],
    [0,          0,          0,          0,          1]
  ], dtype=float)

  S = np.array([S0, S1, S2, S3, Se], dtype=float)
  if S.sum() > 0: S = S / S.sum()

  Y = np.zeros(steps)
```

```
    for n in range(steps):
        Y[n] = (1 - a - d - e + b) * S[3] + b * S[2]
        S = S @ P
    return Y

# ============================================================
# 2. Parameter Fitting Logic
# ============================================================
def fit_vzad_parameters(target_signal):
    steps = len(target_signal)
    def objective(x):
        return np.mean((simulate_VZAD_Y(x, steps) - target_signal)**2)

    cons = ({'type': 'eq', 'fun': lambda x: np.sum(x[4:]) - 1.0})

    # Bounds: S3, Se constrained to 0
    bnds = [(0, 1) for _ in range(7)] + [(0, 0), (0, 0)]

    x0 = [0.1, 0.05, 0.0, 0.0, 0.25, 0.75, 0.0, 0.0, 0.0]

    res = minimize(objective, x0, method='SLSQP', bounds=bnds, constraints=cons, tol=1e-
8)
    return res.x

# ============================================================
# 3. Helpers: Generation & Frequency-Biased EWT
# ============================================================

def generate_test_signals(n_flashes=40, artifact_amp=0.05, artifact_phase=0.0,
                          start_flash=0, artifact_tau=12.0):
    t = np.arange(n_flashes)

    # Truth: Back (d) = 0.02
    true_params = [0.10, 0.05, 0.02, 0.01, 0.25, 0.75, 0, 0, 0]

    A = simulate_VZAD_Y(true_params, n_flashes)

    # Tunable Period-2 Artifact
    B_continuous = artifact_amp * np.cos(np.pi * t + artifact_phase) * np.exp(-t / artifact_tau)

    mask = t >= start_flash
    B = np.zeros_like(t, dtype=float)
    B[mask] = B_continuous[mask]
```

```
    return t, A, B, A + B, true_params

def get_dominant_frequency(signal):
    sig_no_dc = signal - np.mean(signal)
    fft_spectrum = np.fft.rfft(sig_no_dc)
    fft_freqs = np.fft.rfftfreq(len(signal))
    peak_idx = np.argmax(np.abs(fft_spectrum))
    return fft_freqs[peak_idx]

def analyze_ewt_frequency_bias(C, pad_len=12):
    C_padded = np.pad(C, (pad_len, pad_len), mode='reflect')

    params = Default_Params()
    params["detect"] = "localmaxmin"
    params["wavname"] = "littlewood-paley"
    params["N"] = 4
    modes, _, _ = ewt1d(C_padded, params)

    mode_freqs = [get_dominant_frequency(m) for m in modes]
    dist_to_nyquist = [abs(f - 0.5) for f in mode_freqs]
    idx_artifact = int(np.argmin(dist_to_nyquist))

    VZAD_est_padded = np.zeros_like(C_padded)
    for i, m in enumerate(modes):
        if i != idx_artifact:
            VZAD_est_padded += m

    return VZAD_est_padded[pad_len : -pad_len], idx_artifact, mode_freqs[idx_artifact]

# ============================================================
# 4. Scenario Runner
# ============================================================

def run_scenario(scenario_name, n_flashes, amp, phase, start_n, tau):
    print(f"\nRunning: {scenario_name} (N={n_flashes}, Amp={amp}, Tau={tau})")

    # Generate
    t, A_true, B_true, C_mix, true_p = generate_test_signals(n_flashes,
                                        artifact_amp=amp,
                                        artifact_phase=phase,
                                        start_flash=start_n,
                                        artifact_tau=tau)

    # Create plotting time axis (Start at Flash 1)
```

```
    t_plot = t + 1

    # Process
    VZAD_est_ewt, art_idx, art_freq = analyze_ewt_frequency_bias(C_mix, pad_len=12)

    # Fit
    raw_p = fit_vzad_parameters(C_mix)
    ewt_p = fit_vzad_parameters(VZAD_est_ewt)

    # Metrics
    A_raw_fit = simulate_VZAD_Y(raw_p, n_flashes)
    A_ewt_fit = simulate_VZAD_Y(ewt_p, n_flashes)

    # Save Path Handling ## NEW USERS UPDATE THIS
    out_dir = r"C:\Users\Nicholas\OneDrive - Louisiana State
University\Publications\Flash_Oxygen-EWT\output\EWT_v_RAW"

    if not os.path.exists(out_dir):
        os.makedirs(out_dir)

    safe_name = "".join([c if c.isalnum() else "_" for c in scenario_name])

    # =============================================
    # DATA TABLE GENERATION
    # =============================================
    param_labels = ['Miss (a)', 'Double (b)', 'Back (d)', 'Inactive (e)',
                    'S0', 'S1', 'S2', 'S3', 'Se']

    # Construct DataFrame
    df_table = pd.DataFrame({
        'Parameter': param_labels,
        'Truth': true_p,
        'Standard Fit': raw_p,
        'EWT Fit': ewt_p
    })

    # Print to Console
    print("\n" + "="*50)
    print(f"  Comparison Table: {scenario_name}")
    print("="*50)
    pd.set_option('display.float_format', '{:.4f}'.format)
    print(df_table.to_string(index=False))
    print("="*50 + "\n")
```

```
    csv_path = os.path.join(out_dir, f"{safe_name}_parameters.csv")
    df_table.to_csv(csv_path, index=False)
    print(f"[Data] Parameter table saved to: {csv_path}")

    # ==========================================
    # PLOTTING - PUBLICATION STYLE (GRAYSCALE)
    # ==========================================
    plt.rcParams.update({
        'font.size': 14,
        'font.family': 'sans-serif',
        'font.sans-serif': ['Arial', 'Helvetica', 'DejaVu Sans'],
        'axes.linewidth': 1.5,
        'xtick.major.width': 1.5,
        'ytick.major.width': 1.5,
        'xtick.direction': 'in',
        'ytick.direction': 'in',
        'lines.linewidth': 2,
        'legend.frameon': False,
        'legend.fontsize': 12
    })

    fig, ax = plt.subplots(3, 1, figsize=(8, 12))

    # --- Panel A: Signal Components ---
    ax[0].plot(t_plot, A_true, '-', color='gray', linewidth=6, alpha=0.4,
            label='VZAD Simulation', zorder=1)
    ax[0].plot(t_plot, B_true, '--', color='gray', linewidth=2, label='Period-2 simulation',
zorder=2)
    ax[0].plot(t_plot, C_mix, '-', color='black', linewidth=1.5, label='Combined Signal',
zorder=3)

    ax[0].set_ylabel('Amplitude (a.u.)')
    ax[0].set_xlabel('Flash Number')

    # UPDATED: x-axis limits 0 to N+1 for padding
    ax[0].set_xlim(0, n_flashes + 1)

    ax[0].legend(loc='upper right')
    ax[0].text(-0.1, 1.05, 'A', transform=ax[0].transAxes, size=20, weight='bold')

    # --- Panel B: Model Recovery ---
    ax[1].plot(t_plot, A_true, '-', color='gray', linewidth=6, alpha=0.4,
            label='VZAD Simulation', zorder=0)
    ax[1].plot(t_plot, A_raw_fit, '--', color='black', linewidth=2, label='Raw Fit', zorder=2)
```

```
    ax[1].plot(t_plot, A_ewt_fit, '-', color='black', linewidth=2, label='EWT Fit', zorder=2)

    ax[1].set_ylabel('Amplitude (a.u.)')
    ax[1].set_xlabel('Flash Number')

    # UPDATED: x-axis limits 0 to N+1 for padding
    ax[1].set_xlim(0, n_flashes + 1)

    ax[1].legend(loc='upper right')
    ax[1].text(-0.1, 1.05, 'B', transform=ax[1].transAxes, size=20, weight='bold')

    # --- Panel C: Relative Error (Signed) ---
    param_names = [r'$\alpha$', r'$\beta$', r'$\delta$', r'$\epsilon$']
    x = np.arange(4)

    raw_rel_err = (raw_p[:4] - true_p[:4]) / true_p[:4] * 100
    ewt_rel_err = (ewt_p[:4] - true_p[:4]) / true_p[:4] * 100

    width = 0.35

    ax[2].bar(x - width/2, raw_rel_err, width, label='Raw Fit Error',
           color='white', edgecolor='black', hatch='///')
    ax[2].bar(x + width/2, ewt_rel_err, width, label='EWT Fit Error',
           color='black', edgecolor='black')

    ax[2].axhline(0, color='black', linewidth=0.8, linestyle='-')

    ax[2].set_xticks(x)
    ax[2].set_xticklabels(param_names)
    ax[2].set_ylabel("Relative Error (%)")
    ax[2].legend(loc='best')
    ax[2].text(-0.1, 1.05, 'C', transform=ax[2].transAxes, size=20, weight='bold')

    plt.tight_layout()

    save_path = os.path.join(out_dir, f"{safe_name}.png")

    print(f"Saving to: {save_path}")
    plt.savefig(save_path, dpi=300)
    plt.show()

# =====================================================================
# MAIN
# =====================================================================
```

```
if __name__ == "__main__":

    # Scenario: Moderate Interference
    scenarios = [
        ("Moderate Interference", 24, 0.05, 0.0, 0, 12.0),
    ]

    for sc in scenarios:
        run_scenario(*sc)
```

Appendix S2. Spectral isolation and quinone model script for Figures 2-4 written in Python.

```
#!/usr/bin/env python3
# -*- coding: utf-8 -*-
"""
Modified for Windows Compatibility
@nf gemini3
VZAD Model + EWT Analysis on Real Data (Normalized)
"""

from __future__ import annotations

from pathlib import Path
import re
import sys

import numpy as np
import matplotlib.pyplot as plt
from scipy.optimize import minimize
import pandas as pd

# ============================================================
# WINDOWS INSTALLATION NOTE FOR pyewt:
# If 'pip install pyewt' fails on Windows, you may need to install
# "Microsoft C++ Build Tools" first, or look for a pre-compiled wheel.
# ============================================================
try:
   from pyewt.defaultparams import Default_Params
   from pyewt.ewt1d import ewt1d, iewt1d
except ImportError:
   print("Error: pyewt not found. Please install via: pip install pyewt")
   # On Windows, if pip fails, ensure you have C++ Build Tools installed.
   sys.exit(1)


# ============================================================
# 0. Paths / Config (WINDOWS ADJUSTED)
# ============================================================

# 1. Get the current user's home directory (e.g., C:\Users\Nicholas)
USER_HOME = Path.home()

# 2. Define the Base Directory for your OneDrive.
```

```
# CHECK THIS
ONEDRIVE_FOLDER = USER_HOME / "OneDrive - Louisiana State University"

# 3. Construct the full paths
DEFAULT_FLASH_CSV = ONEDRIVE_FOLDER / "Publications" / "Flash_Oxygen-EWT" /
"input" / "flash_seqs.csv"
DEFAULT_OUTPUT_DIR = ONEDRIVE_FOLDER / "Publications" / "Flash_Oxygen-EWT" /
"output" / "Empirical_fits"


# ============================================================
# 1. Experimental data I/O + normalization
# ============================================================
def list_available_sequences(csv_path: Path = DEFAULT_FLASH_CSV) -> list[str]:
    """
    Returns column names that look like usable sequences.
    """
    if not csv_path.exists():
        raise FileNotFoundError(f"Could not find input CSV at:\n{csv_path}\nPlease check the
ONEDRIVE_FOLDER path in the script.")

    df = pd.read_csv(csv_path, encoding='utf-8')

    ignore = {"n", "flash", "flash#", "flash_num", "index", "time", "t"}
    seqs: list[str] = []

    for c in df.columns:
        if c is None:
            continue
        if str(c).strip().lower() in ignore:
            continue

        s = pd.to_numeric(df[c], errors="coerce")
        if s.notna().sum() == 0:
            continue

        seqs.append(c)

    return seqs


def get_experimental_data(sequence: str, csv_path: Path = DEFAULT_FLASH_CSV) ->
np.ndarray:
    """
```

```
    Load one sequence column from flash_seqs.csv by name.
    """
    if not csv_path.exists():
        raise FileNotFoundError(f"Input file not found: {csv_path}")

    df = pd.read_csv(csv_path, encoding='utf-8')

    if sequence not in df.columns:
        avail = list_available_sequences(csv_path)
        raise ValueError(
            f"Sequence '{sequence}' not found in {csv_path}.\n"
            f"Available sequences: {avail}"
        )

    y = pd.to_numeric(df[sequence], errors="coerce").dropna().to_numpy(dtype=float)

    if y.size == 0:
        raise ValueError(f"Sequence '{sequence}' contains no numeric data after parsing.")

    return y


def normalize_data_to_model(y: np.ndarray, steady_state_value: float = 0.25, tail_n: int = 4)
-> np.ndarray:
    """
    Scales experimental data so the steady-state tail matches the model's
steady_state_value.
    """
    if y.size < tail_n:
        raise ValueError(f"Need at least {tail_n} flashes to normalize, got {y.size}.")

    tail_avg = float(np.mean(y[-tail_n:]))
    if tail_avg == 0:
        raise ValueError("Tail average is zero; cannot normalize.")
    scaling_factor = steady_state_value / tail_avg
    return y * scaling_factor


# ============================================================
# 2. VZAD Kok Model Logic
# ============================================================


def simulate_VZAD_Y(params: np.ndarray, steps: int) -> np.ndarray:
```

```
    a, b, d, e, S0, S1, S2, S3, Se = params

    # Matrix P: Rows = FROM state, Cols = TO state
    # Rows sum to 1.
    P = np.array([
        # S0 -> S0 (miss), S1 (hit), S2 (double)
        [a,          1 - a - b,    b,          0,          0],
        # S1 -> S1 (miss), S2 (hit), S3 (double)
        [0,          a,          1 - a - b,    b,          0],
        # S2 -> S0 (double), S1 (back), S2 (miss), S3 (hit)
        [b,          d,          a,          1 - a - b - d, 0],
        # S3 -> S0 (hit), S1 (double), S2 (back), S3 (miss), Se (inact)
        [1 - a - b - d - e, b,       d,          a,          e],
        # Se -> Se (absorbing)
        [0,          0,          0,          0,          1]
    ], dtype=float)

    S = np.array([S0, S1, S2, S3, Se], dtype=float)
    if S.sum() > 0:
        S = S / S.sum()

    Y = np.zeros(steps, dtype=float)

    for i in range(steps):
        # Calculate Yield based on CURRENT state before transition
        # Oxygen evolves on S3->S0 (single hit) and S3->S1 (double hit)
        # Oxygen also evolves on S2->S0 (double hit)

        # Contribution from S3: (1-a-b-d-e) + b = (1-a-d-e)
        # Contribution from S2: b
        Y[i] = (1 - a - d - e) * S[3] + b * S[2]

        # Update State: S_new = S_old * P
        S = S @ P

    return Y


# ============================================================
# 3. Fitting Logic
# ============================================================
def fit_vzad_parameters(target_signal: np.ndarray, s2_limit: float = 0.15, s3_limit: float =
0.0) -> np.ndarray:
    steps = len(target_signal)
```

```
    def objective(x: np.ndarray) -> float:
        return float(np.mean((simulate_VZAD_Y(x, steps) - target_signal) ** 2))

    cons = (
        {"type": "eq",   "fun": lambda x: np.sum(x[4:]) - 1.0},
        {"type": "ineq", "fun": lambda x: s2_limit - x[6]},
        {"type": "ineq", "fun": lambda x: s3_limit - x[7]},
    )

    bnds = [(0.0, 1.0) for _ in range(9)]
    x0 = np.array([0.1, 0.05, 0.0, 0.0, 0.1, 0.9, 0.0, 0.0, 0.0], dtype=float)

    res = minimize(objective, x0, method="SLSQP", bounds=bnds, constraints=cons, tol=1e-
8)
    if not res.success:
        print(f"[WARN] Fit did not fully converge: {res.message}")
    return np.array(res.x, dtype=float)


# ============================================================
# 4. Robust EWT
# ============================================================
def get_dominant_frequency(signal: np.ndarray) -> float:
    sig_no_dc = signal - np.mean(signal)
    fft_spectrum = np.fft.rfft(sig_no_dc)
    fft_freqs = np.fft.rfftfreq(len(signal))
    peak_idx = int(np.argmax(np.abs(fft_spectrum)))
    return float(fft_freqs[peak_idx])


def analyze_ewt_robust(C: np.ndarray, pad_len: int = 12):
    C_padded = np.pad(C, (pad_len, pad_len), mode="reflect")

    params = Default_Params()
    params["detect"] = "localmaxmin"
    params["wavname"] = "littlewood-paley"
    params["N"] = 4

    modes, _, _ = ewt1d(C_padded, params)

    mode_freqs = [get_dominant_frequency(m) for m in modes]
    dist_to_nyquist = [abs(f - 0.5) for f in mode_freqs]
    idx_artifact = int(np.argmin(dist_to_nyquist))
```

```
    VZAD_est_padded = np.zeros_like(C_padded)
    for i, m in enumerate(modes):
        if i != idx_artifact:
            VZAD_est_padded += m

    VZAD_est = VZAD_est_padded[pad_len:-pad_len]
    Artifact_est = modes[idx_artifact][pad_len:-pad_len]

    return VZAD_est, Artifact_est, idx_artifact


# ============================================================
# 5. q(n) definition
# ============================================================
def q_of_n(n: np.ndarray, params: np.ndarray):
    a, b, d, e, S0, S1, S2, S3, Se = params
    g = 1.0 - a - b - d - e
    qn = 0.5 * (1.0 + (1.0 - 2.0 * g) ** n) * (S0 - S1 + S2 - S3)
    return qn, float(g)


# ============================================================
# 6. One-run wrapper
# ============================================================
def _safe_name(s: str) -> str:
    # This regex is safe for Windows filenames (no : < > " / \ | ? *)
    return re.sub(r"[^A-Za-z0-9_.-]+", "_", s.strip())


def run_workflow_for_sequence(
    sequence: str,
    csv_path: Path = DEFAULT_FLASH_CSV,
    output_dir: Path = DEFAULT_OUTPUT_DIR,
    steady_state_value: float = 0.25,
    tail_n: int = 4,
    pad_len: int = 12,
    s2_limit: float = 0.15,
    s3_limit: float = 0.0,
):
    # Load + normalize
    y_raw = get_experimental_data(sequence, csv_path=csv_path)
    y_exp = normalize_data_to_model(y_raw, steady_state_value=steady_state_value,
tail_n=tail_n)
```

```
t = np.arange(1, len(y_exp) + 1)

print(f"\n=== {sequence} ===")
print("Processing EWT (Padded + Freq Bias)...")
y_ewt, artifact_est, _ = analyze_ewt_robust(y_exp, pad_len=pad_len)

print("Fitting Models...")
raw_p = fit_vzad_parameters(y_exp, s2_limit=s2_limit, s3_limit=s3_limit)
ewt_p = fit_vzad_parameters(y_ewt, s2_limit=s2_limit, s3_limit=s3_limit)

N = len(t)
y_fit_raw = simulate_VZAD_Y(raw_p, N)
y_fit_ewt = simulate_VZAD_Y(ewt_p, N)

n = t.astype(float)
qn, g_val = q_of_n(n, ewt_p)

art = artifact_est - np.mean(artifact_est)
qn0 = qn - np.mean(qn)
art_rms = np.sqrt(np.mean(art ** 2)) + 1e-12
q_rms = np.sqrt(np.mean(qn0 ** 2)) + 1e-12
qn_scaled = qn0 * (art_rms / q_rms)

param_names = ['a (miss)', 'b (dbl)', 'd (back)', 'e (inact)', 'S0', 'S1', 'S2', 'S3', 'Se']
df = pd.DataFrame({'Parameter': param_names, 'Raw': raw_p, 'EWT': ewt_p})
df['Difference'] = df['Raw'] - df['EWT']
pd.set_option('display.float_format', '{:.4f}'.format)

print(df.to_string(index=False))
print(f"q(n) uses g = 1-a-b-d-e = {g_val:.4f}")

# ============================================
#  SAVE TO CSV
# ============================================
csv_outpath = output_dir / f"{_safe_name(sequence)}_parameters.csv"
df.to_csv(csv_outpath, index=False)
print(f"[saved csv] {csv_outpath}")
# ============================================

# ============================================
# PLOTTING - PUBLICATION STYLE (GRAYSCALE)
# ============================================
```

```
# 1. Global Style Settings
plt.rcParams.update({
    'font.size': 14,
    'font.family': 'sans-serif',
    'font.sans-serif': ['Arial', 'Helvetica', 'DejaVu Sans'],
    'axes.linewidth': 1.5,
    'xtick.major.width': 1.5,
    'ytick.major.width': 1.5,
    'xtick.direction': 'in',
    'ytick.direction': 'in',
    'lines.linewidth': 2,
    'legend.frameon': False,
    'legend.fontsize': 12
})

fig, ax = plt.subplots(3, 1, figsize=(8, 12), sharex=True)

# --- Panel A: Separation ---
# Raw Data (Combined Style): Thick Gray Line (Background) + Open Markers (Foreground)
ax[0].plot(t, y_exp, '-', color='#cccccc', linewidth=6, zorder=0) # Background line
ax[0].plot(t, y_exp, 'o', color='black', markerfacecolor='white',
        markeredgewidth=1.5, markersize=6, label='Raw Data', zorder=2) # Foreground
markers

# V-Space (EWT Trend): Solid Black line
ax[0].plot(t, y_ewt, '-', color='black', linewidth=2, label='Period-4 Component', zorder=1)

# W-Component (Artifact): Gray Dashed line
ax[0].plot(t, artifact_est, '--', color='gray', linewidth=2, label='Period-2 Component',
zorder=1)

ax[0].set_ylabel('Yield ($Y_n$)')
ax[0].legend(loc='upper right')
ax[0].text(-0.1, 1.05, 'A', transform=ax[0].transAxes, size=20, weight='bold')

# --- Panel B: Model Fits ---
# Raw Data (Combined Style): Thick Gray Line (Background) + Open Markers (Foreground)
ax[1].plot(t, y_exp, '-', color='#cccccc', linewidth=6, zorder=0) # Background line
ax[1].plot(t, y_exp, 'o', color='black', markerfacecolor='white',
        markeredgewidth=1.5, markersize=6, label='Raw Data', zorder=3) # Foreground
markers (on top of fits)

# Raw Fit: Black Dotted line
ax[1].plot(t, y_fit_raw, ':', color='black', linewidth=2.5, label='Raw Fit', zorder=2)
```

```
    # EWT Fit: Black Solid line (thinner)
    ax[1].plot(t, y_fit_ewt, '-', color='black', linewidth=1.5, label='EWT Fit', zorder=2)

    ax[1].set_ylabel('Yield ($Y_n$)')
    ax[1].legend(loc='upper right')
    ax[1].text(-0.1, 1.05, 'B', transform=ax[1].transAxes, size=20, weight='bold')

    # --- Panel C: Parity (Q_B vs W) ---
    # W-Component (Isolated P2): Gray Dashed Line + Markers
    ax[2].plot(t, art, 'o--', color='gray', markersize=5, linewidth=2, label='Period-2
Component')

    # Predicted Q_B: Black Solid line
    ax[2].plot(t, qn_scaled, '-', color='black', linewidth=2, label='Predicted $Q_B^-$')

    # Zero line
    ax[2].axhline(0, color='black', linewidth=0.5, zorder=0)

    ax[2].set_ylabel('Amplitude (a.u.)')
    ax[2].set_xlabel('Flash Number')
    ax[2].legend(loc='upper right')
    ax[2].text(-0.1, 1.05, 'C', transform=ax[2].transAxes, size=20, weight='bold')

    plt.tight_layout()
    plt.subplots_adjust(hspace=0.25)

    output_dir.mkdir(parents=True, exist_ok=True)
    outpath = output_dir / f"{_safe_name(sequence)}_fit.png"
    plt.savefig(outpath, dpi=300) # Slightly lower DPI for faster Windows rendering
    plt.show()
    print(f"[saved] {outpath}")

    return {
        "sequence": sequence,
        "y_raw": y_raw,
        "y_exp": y_exp,
        "t": t,
        "y_ewt": y_ewt,
        "artifact_est": artifact_est,
        "raw_p": raw_p,
        "ewt_p": ewt_p,
        "g": g_val,
        "qn": qn,
        "qn_scaled": qn_scaled,
```

```
        "params_df": df,
        "figure_path": outpath,
    }


# ============================================================
# MAIN
# ============================================================
if __name__ == "__main__":

    # Run whichever sequences you want
    sequences_to_run = ["forbush", "brandon", 'Joliot Short']

    print(f"Reading: {DEFAULT_FLASH_CSV}")

    # Catch errors early if paths are wrong
    try:
        available = list_available_sequences(DEFAULT_FLASH_CSV)
        print("Available sequences:", available)

        results = {}
        for seq in sequences_to_run:
            results[seq] = run_workflow_for_sequence(seq, csv_path=DEFAULT_FLASH_CSV)

    except FileNotFoundError as e:
        print("\n[PATH ERROR]")
        print(e)
        print("\nTip: Edit the 'ONEDRIVE_FOLDER' variable at the top of the script.")
```

Appendix S3. Updated version of VZAD written in Python.

```
import numpy as np
import pandas as pd
import matplotlib.pyplot as plt
import math
from scipy.optimize import differential_evolution
from scipy.interpolate import CubicSpline
# Load the data
data = pd.read_csv("0xCatalaseEWT.txt", delimiter="\t", header=None, names=["Step",
"Y_obs"])
steps = len(data)
# Define the function to run the Markov process and compute Y values
def simulate_Y(params):
a, b, d, e, S0, S1, S2, S3, Se = params
# Transition probability matrix
P = np.array([
[a, 0, (b - b*e), (1 - b - d - a - e), 0],
[(1 - a - b), a, d, (b - b*e), 0],
[b, (1 - a - b), a, d, 0],
[0, b, (1 - b - d - a), a, 0],
[0, 0, b*e, (e + b*e), 1]])
# Initial state vector
S = np.array([S0, S1, S2, S3, Se])
Y_values = []
Y_prev = (1 - a - d - e) * S[3] + b * S[2] # Initial Y using S2, S3
for _ in range(steps):
```

```
S = P @ S # Update state populations
Y_values.append(Y_prev) # Use previous step's Y
Y_prev = (1 - a - d - e) * S[3] + b * S[2] # Compute next Y
return np.array(Y_values)
# Define the loss function (mean squared error)
def loss_function(params):
Y_simulated = simulate_Y(params)
return np.mean((Y_simulated - data["Y_obs"])**2)
# Initial guesses for parameters
initial_guess = [0.05, 0.05, 0.05, 0.02, 2, 2, 0.5, 0.5, 0.1]
# Bounds for parameters
bounds = [(0, 0.4), (0, 0.4), (0, 0.4), (0, 0.4), (0, 10), (0, 10), (0, 10), (0, 10), (0, 10)]
# Run optimization using Differential Evolution
result = differential_evolution(loss_function, bounds, strategy='best1bin', maxiter=1000,
popsize=15)
# Get optimized parameters
optimized_params = result.x
optimized_Y = simulate_Y(optimized_params)
# Compute Theoretical Period
def lambda_4(a, b, d, e):
term1 = complex(a - b, -(1 - a - b - d))
term2 = e * complex(-2*b**3 + 2*b*a**2 + 4*a*b**2 - 6*a*b + a*b*d + 4*b - 2*b*d - 6*b**2 +
b**2*d,
2 + 2*a**2 - 2*b**2 - 2*d + d**2 - 4*a + 4*a*b + 2*a*d - 4*b + 2*b*d) \
/ (2 * complex(1 + 4*b**3 + a**2 - 2*a + 4*a*b**2 + 2*a*b - 2*b - 5*b**2 + 2*b**2*d,
4*b**2 + 4*a*b - 4*b + 4*b*d))
```

```
return term1 + term2

phase_value = math.acos(lambda_4(optimized_params[0], optimized_params[1],

optimized_params[2], optimized_params[3]).real /

abs(lambda_4(optimized_params[0], optimized_params[1],

optimized_params[2], optimized_params[3])))

theor_period = 2 * math.pi / phase_value

# Plot observed vs. fitted Y values and residuals

plt.figure(figsize=(12, 6))

plt.plot(data["Step"], data["Y_obs"], label="Observed Data", marker="o")

plt.plot(data["Step"], optimized_Y, label="Fitted Model", linestyle="--")

plt.plot(data["Step"], data["Y_obs"] - optimized_Y, label="Residuals", color='red',
marker="o",

linestyle=":")

plt.axhline(0, color='black', linestyle='--')

plt.xlabel("Step")

plt.ylabel("Y Value & Residuals")

plt.title("Observed vs. Fitted Y Values & Residuals")

plt.legend()

plt.grid(True)

plt.show()

# Plot FFT Analysis

plt.figure(figsize=(12, 6))

plt.plot(filtered_frequencies, filtered_magnitudes, label="FFT of Raw Data", marker="o")

plt.plot(smoothed_frequencies, smoothed_magnitudes, label="Splined Function",
linestyle="--")

plt.axvline(x=max_freq, color='red', linestyle='--', label=f'Max Frequency: {max_freq:.4f}')

plt.xlabel("Frequency")
```

```
plt.ylabel("Magnitude")
plt.title("FFT Analysis of Raw Data")
plt.legend()
plt.grid(True)
plt.show()
# Perform Fast Fourier Transform (FFT) on observed data
y_fft = np.fft.fft(data["Y_obs"])
n = len(data)
frequencies = np.fft.fftfreq(n)
# Keep only frequencies in the range [0.05, 0.5]
mask = (frequencies > 0.05) & (frequencies < 0.5)
filtered_frequencies = frequencies[mask]
filtered_magnitudes = np.abs(y_fft[mask])
# Apply cubic spline smoothing
spline = CubicSpline(filtered_frequencies, filtered_magnitudes)
smoothed_frequencies = np.linspace(0.05, 0.5, 500)
smoothed_magnitudes = spline(smoothed_frequencies)
# Find frequency with maximum magnitude
max_freq = smoothed_frequencies[np.argmax(smoothed_magnitudes)]
fft_period = 1 / max_freq
# Convert all columns to float before padding
data["Step"] = data["Step"].astype(float)
data["Y_obs"] = data["Y_obs"].astype(float)
# Ensure max_length is correctly computed
max_length = max(len(data["Step"]), len(optimized_Y), len(filtered_frequencies),
len(smoothed_frequencies))
```

```
# Create export data dictionary with corrected padding
export_data = {
"Step": np.pad(data["Step"], (0, max_length - len(data["Step"])), constant_values=np.nan),
"Observed Y": np.pad(data["Y_obs"], (0, max_length - len(data["Y_obs"])),
constant_values=np.nan),
"Fitted Y": np.pad(optimized_Y, (0, max_length - len(optimized_Y)),
constant_values=np.nan),
"Residuals": np.pad(data["Y_obs"] - optimized_Y, (0, max_length - len(optimized_Y)),
constant_values=np.nan),
"FFT Frequency": np.pad(filtered_frequencies, (0, max_length - len(filtered_frequencies)),
constant_values=np.nan),
"FFT Magnitude": np.pad(filtered_magnitudes, (0, max_length - len(filtered_magnitudes)),
constant_values=np.nan),
"Splined Frequency": np.pad(smoothed_frequencies, (0, max_length -
len(smoothed_frequencies)), constant_values=np.nan),
"Splined Magnitude": np.pad(smoothed_magnitudes, (0, max_length -
len(smoothed_magnitudes)), constant_values=np.nan),
}
# Normalize S0-S3
S_total = optimized_params[4] + optimized_params[5] + optimized_params[6] +
optimized_params[7]
S0_norm = optimized_params[4] / S_total
S1_norm = optimized_params[5] / S_total
S2_norm = optimized_params[6] / S_total
S3_norm = optimized_params[7] / S_total
# Prepare parameters for export
```

```
parameters = {
"Parameter": ["a", "b", "d", "e", "S0(norm)", "S1(norm)", "S2(norm)", "S3(norm)", "Se", "Theor
Period", "FFT Period"],
"Value": [optimized_params[0], optimized_params[1], optimized_params[2],
optimized_params[3],
S0_norm, S1_norm, S2_norm, S3_norm, optimized_params[8], theor_period, fft_period]}
parameters_df = pd.DataFrame(parameters)
# Append parameter data to export_data
for i, row in parameters_df.iterrows():
export_data[row["Parameter"]] = [row["Value"]] + [np.nan] * (max_length - 1)
# Export results to a CSV file
csv_export_path = "Optimized_Markov_Fit_DE.csv"
export_df = pd.DataFrame(export_data)
export_df.to_csv(csv_export_path, index=False)
print(f"Exported results saved to {csv_export_path}")
```
